# Yeast Starter Culture Identification to Produce of Red Wines with Enhanced Antioxidant Content

**DOI:** 10.3390/foods13020312

**Published:** 2024-01-18

**Authors:** Giuseppe Romano, Marco Taurino, Carmela Gerardi, Maria Tufariello, Marcello Lenucci, Francesco Grieco

**Affiliations:** 1 National Research Council, Institute of Sciences of Food Production (ISPA), Via Prov. Lecce-Monteroni, 73100 Lecce, Italy; giuseppe.romano@ispa.cnr.it (G.R.); marco.taurino@ispa.cnr.it (M.T.); carmela.gerardi@ispa.cnr.it (C.G.); maria.tufariello@ispa.cnr.it (M.T.); 2 Department of Biological and Environmental Sciences and Technologies, University of Salento, Via Prov. Lecce-Monteroni, 73100 Lecce, Italy; marcello.lenucci@unisalento.it

**Keywords:** autochthonous yeast, *Saccharomyces cerevisiae*, wine polyphenols

## Abstract

Grape variety, quality, geographic origins and phytopathology can influence the amount of polyphenols that accumulate in grape tissues. Polyphenols in wine not only shape their organoleptic characteristics but also significantly contribute to the positive impact that this beverage has on human health. However, during the winemaking process, the total polyphenol content is substantially reduced due to the adsorption onto yeast wall polymers and subsequent lees separation. Despite this, limited information is available regarding the influence of the yeast starter strain on the polyphenolic profile of wine. To address this issue, a population consisting of 136 Saccharomyces cerevisiae strains was analyzed to identify those with a diminished ability to adsorb polyphenols. Firstly, the reduction in concentration of polyphenolic compounds associated to each strain was studied by assaying Total Phenolic Content (TPC) and Trolox Equivalent Antioxidant Capacity (TEAC) in the wines produced by micro-scale must fermentation. A total of 29 strains exhibiting a TPC and TEAC reduction ≤ 50%, when compared to that detected in the utilized grape must were identified and the nine most-promising strains were further validated by larger-scale vinification. Physico-chemical analyses of the resulting wines led to the identification of four strains, namely ITEM6920, ITEM9500, ITEM9507 and ITEM9508 which showed, compared to the control wine, a TPC and TEAC reduction ≤ 20 in the produced wines. They were denoted by a significant (*p* < 0.05) increased amount of anthocyanin, quercetin and trans-coutaric acid, minimal volatile acidity (<0.2 g/L), absence of undesirable metabolites and a well-balanced volatile profile. As far as we know, this investigation represents the first clonal selection of yeast strains aimed at the identifying “functional” fermentation starters, thereby enabling the production of regional wines with enriched polyphenolic content.

## 1. Introduction

Wine shows a complex chemical profile marked by different classes of metabolites that influence its nutritional, physiological, and aromatic value. Some of these compounds, known as varietal molecules, originate from grapes, while others are produced during the fermentation and wine aging processes. Phenolic compounds emerge as a key factor in the quality of wines, especially red ones. They play a dual role, not only enhancing wine quality properties like colour, clarity, taste structure, and resistance to oxidation but also contributing to the prevention of chronic diseases and promoting healthy aging. These physiological effects are particularly associated with flavonoid and stilbene contents, including molecules such as quercetin, catechins, resveratrol and trans-resveratrol [1,2]. The amount of the different polyphenolic classes is an important index of the nutritional quality of the product, since wine stands out as one of the main sources of antioxidants in the Mediterranean diet [3]. The polyphenolic extracts of red wine are a complex mixture of structurally different compounds, some of which exhibit important biological activities, such as the prevention of cardiovascular diseases [4,5], inhibition of inflammatory processes, and protection against certain cancers [6,7]. However, the specific roles of individual polyphenols, whether flavonoids or non-flavonoids, remain not entirely clear, and are often attributed to their synergistic action. Several factors influence the phenolic content of wine, with some of the most important ones being the physical or enzymatic interventions during maceration and the yeast strain used in the fermentation process. Currently, ongoing research delves into the mechanisms through which yeast influences the colour and polyphenolic compound content of wine. Three modes of interaction between yeast and the polyphenolic component have already been described. The first mechanism involves the adsorption of polyphenols on the cell wall of yeasts. However, although yeast has been shown as a factor capable of inducing the loss of part of polyphenols in wines, it remains unclear whether anthocyanin adsorption on the cell wall is the only mechanism at play.

The cell wall of *S. cerevisiae* is characterised by outward-facing mannoproteins bound to oligosaccharides, glucans, and chitin [8]. The different polarities of these cell wall components affect the yeast’s capacity to absorb and retain certain classes of molecules, such as polyphenols, volatile compounds, and fatty acids [9]. The adsorption of molecules onto the yeast cell wall is further influenced by its porosity, with greater interstitial spaces providing an increased surface area that favours adsorption [10]. During alcoholic fermentation, the substantial biomass generated leads to a significant proportion of polyphenols being adsorbed onto the cell walls and subsequently removed from the wine along with the lees. It is plausible that different yeast strains have distinct cell wall composition, influencing the varying degrees of adsorption of phenolic compounds. Moreover, the potential for certain strains to demonstrate distinct adsorption patterns, selectively interacting with specific classes of polyphenols, cannot be ruled out. 

Another form of interaction is associated with the enzymatic activity of β-glucosidase, which is released by the yeasts themselves [11]. Most of the anthocyanins in wine exist in a glycosylated form, i.e., bound to a sugar. In this state, they are much less susceptible to chemical or enzymatic oxidation. Therefore, the action of β-glucosidase, which generates the respective aglycones (anthocyanidins) in the wine, can promote their removal during winemaking [12]. 

Finally, some yeast strains release polysaccharides capable of binding with polyphenols, forming stable complexes over time. The presence of these complexes is directly related to the sensations of volume and roundness in the mouth, as well as the stability of wine over time. Research has demonstrated that several yeast metabolites, including pyruvic acid, can react with anthocyanins in grapes to form stable pigmentation and contribute to the aging of red wines.

Unfortunately, the absorption of these molecules onto yeast cell walls, and consequently, their decrease in the produced wine, poses a significant challenge in the fermentation process. This issue has garnered attention from researchers and winemakers. In this light, to prevent or reduce the loss of bioactive molecules through absorption, we studied the absorption capacity of some indigenous yeast strains selected in the Apulia region (Southern Italy).

The use of autochthonous selected yeasts initially gained popularity in white wine production and later extended to the crafting of red wines [13]. In recent years, several studies have underlined the pivotal role played by the microbiota associated with the “terroir” where a particular grape cultivar is cultivated. This microbiota imparts unique sensory properties to the resulting wine [14,15]. 

The employment of selected autochthonous yeast strains emerges as a powerful tool to enhance the organoleptic and sensory attributes of distinctive regional wines, establishing a stronger connection between these wines and their terroir [16].

The natural biodiversity of Apulian autochthonous yeast strains has been widely investigated [17,18,19,20,21,22,23,24,25]. It therefore appeared important to exploit our knowledge to identify yeast strains able to enhance the phenolic compound content and, consequently, the functional properties of produced wines.

As a first step, we proceeded with the characterization of a population of selected starter cultures according to their ability to minimize the reduction in the concentration of polyphenolic compounds during a micro-scale must fermentation. Then, the performances of the most effective strains were further validated through larger-scale vinification, and the resulting wines were analysed for their polyphenolic profiles. To the best of our knowledge, this study represents the first clonal selection of yeast strains directed towards the production of regional wines enriched in their polyphenolic content, to be used in the near future for the development of “functional” fermentation starter.

## 2. Materials and Methods

### 2.1. Yeast Strains

Yeast strains used in the present study were deposited in Agro-Food Microbial Culture Collection of ISPA (http://www.ispacnr.it/collezioni-microbiche, accessed on 18 December 2023). Yeasts were cultured in YPD broth (10 g/L yeast extract, 20 g/L peptone, 20 g/L glucose, 20 g/L agar) at 28 °C for 24 h, and maintained at −80 °C in glycerol 50%. Yeast populations were sampled at the end of the alcoholic fermentation process. Yeast total genomic DNA was extracted according to De Benedictis et al. [17] and isolates were genetically distinguished at strain level by inter-delta typing [26].

### 2.2. Vinifications

Primitivo (*Vitis vinifera*) grapes were sampled in a vineyard, with deep, clay-limestone soil, located in Cutrofiano (Lecce, Apulia, southern Italy), an area with a temperate climate. The vineyard was organically managed, fertilized with organic manure and without the use of fungicides.

The strains were firstly tested by a microfermentation assay in Primitivo grapes must (sugars 190 g/L, 20° Brix, pH 3.31) Then, the selected yeast strains were further assayed by inoculating two liters of Primitivo grape must (sugars 206 g/L, 21° Brix, pH 3.25). 

Both grape musts were previously added with 100 mg/L potassium metabisulphite and the alcoholic fermentation was carried out in triplicate as described by Grieco et al. [27]. The samples of fermented must were stored at −20 °C until required for analysis. Each fermentation experiment was carried out by performing three simultaneous independent repetitions. The commercial starter CM was used as control since it was the most used strain by the winemakers in the sampled area. WineScanTM Flex (FOSS Italia S.r.l., Padova, Italy) was used to determine the total acidity and volatile compounds as well as the concentration of ethanol, reducing sugars, malic and lactic acids, and glycerol. Samples were centrifuged at 8000× *g* for 10 min and then analysed. The analyses were performed in triplicate.

### 2.3. Total Polyphenols Content

The total amount of polyphenols was measured by the optimized Folin–Ciocalteu method [28]. The total phenolic content in wine extracts was determined by measuring the absorbance at 765 nm according to the Folin–Ciocalteu colorimetric method. Results were expressed as milligram gallic acid equivalents per liter (mg GAEs/L).

### 2.4. Total Anthocyanin Content

The total content of anthocyanins was determined on wine samples using the pH differential method [29]. The wine samples were mixed using the appropriate dilution factor, with two different solutions to obtain different pH values, prepared as previously described [30]: pH 1.0 potassium chloride buffer (0.025 M KCl) and pH 4.5 sodium acetate buffer (0.4 M CH_3_CO_2_Na·3H_2_O). After 15 min incubation at room temperature, the absorbance of the samples was measured at 520 nm and 700 nm (Shimadzu UV-1800, spectrophotometer, Kyoto, Japan). The total content of anthocyanins, expressed as oenin equivalents, was calculated according to the formula described in Lee et al. [29] using a MW (molecular weight) = 493.5 g/mol for malvidin-3-glucoside and a molar extinction coefficient ε = 2690 L mol^–1^ ∙ cm^–1^.

### 2.5. TEAC Antioxidant Capacity Determination

The Trolox equivalent antioxidant capacity (TEAC) assay is based on the scavenging ability of antioxidants to quench the radical cationic activity of 2,20-azinobis (3-ethylbenzoithiazolone 6-sulphonate) (ABTS^+^). The assay was performed as previously described [31] with some modifications. To generate the ABTS^+^ radical cation, ABTS was dissolved in water (7 mM) and incubated with 2.45 mM potassium persulfate (final concentration) in the dark at room temperature for 12–16 h before use. For the calibration curve, the ABTS^+^ solution was diluted with water to an absorbance value of 0.70 (±0.02) at 734 nm and mixed with 20 µL of Trolox standard solutions (from 0 to 25 µM). The assay was performed with extracts from wine, and absorbance was determined at 734 nm. Values were expressed as µmol Trolox equivalents (TE)/L.

### 2.6. Determination of Polyphenolic Profile of Wines

A reversed-phase HPLC analytical method was used for the analysis of polyphenolic compounds. The apparatus was an Agilent-1100 liquid chromatograph (Agilent Technologies, Santa Clara, CA, USA) equipped with a DAD detector (Agilent 1260 Infinity) and the separation was performed on a C18 column (5 µm UltraSphere 80 Å, 4.6 i.d. × 250 mm length) following the conditions described by Gerardi et al. [32]. Chromatograms were acquired at 520, 280, 320, 370 and 306 nm. The following reference compounds (purchased from Sigma-Aldrich, Saint Louis, MO, USA) were used, each with its retention time indicated in parentheses: quercetin (37.17 min), gallic acid (5.57 min), catechin (12.09 min), oenin (27.60 min), trans-resveratrol (35.74 min), caftaric acid (10.42 min), coutaric acid (14.43 min).

Identification of compounds was based on the comparison of peak retention time with the retention time and UV–vis spectra of pure standards while quantification was performed by adopting the external standard method.

### 2.7. Volatile Profile 

The extraction of volatile compounds was performed by a solid phase microextraction in combination with gas chromatography coupled to mass spectrometry (SPME-GC/MS). According to Palombi et al. [33], 100 μL of internal standard solution (IS, 4-methyl-2-pentanol, 300 mg L^−1^) was added to a volume of 5 mL of wine in a 20 mL headspace vial (Alltech Corp., Deerfield, IL, USA). A 50/30 DVB-CAR-PDMS solid phase microextraction (SPME) fiber (Supelco, Bellofonte, PA, USA) was inserted into the vial and let to adsorb volatiles for 30 min at 40 °C and then transferred to the injector port (250 °C) where desorption occurred in 2 min. Splitless mode was selected as injection mode. GC-MS analyses were performed on a GC 6890 (Agilent Technologies, Santa Clara, CA, USA) coupled to an Agilent MSD 5973 Network detector using a HP-INNOWAX capillary column (60 m × 0.25 mm, 0.25 μm, J & W Scientific Inc., Folsom, CA, USA) as reported by Tufariello et al. [34]. The annotation of the volatile compounds was achieved by comparing mass spectra with those of the data system library (NIST 98, *p* > 90%), with the retention data of commercially available standards and MS data reported in the literature. Concentration of each volatile compound was assessed by the internal standard method [35,36].

### 2.8. Statistical Analysis

Values reported, represent the mean ± standard deviation of three independent replicates. The Analysis of Variance (ANOVA) and Tukey’s post hoc method were applied to highlight significant differences between each yeast strain versus control for chemical parameters (*p* value < 0.05). Principal Component Analysis (PCA) was applied to separate yeast strains tested according to the results of the chemical analyses. Tukey’s post hoc method was carried out for assessing significant differences between means (*p* < 0.05) by using SigmaStat software Version 3.1 (Jandel Corp., Erkrath, Germany). The principal component analysis of volatile compounds was carried out using the Statistica 6.0 software package.

## 3. Results

A laboratory-scale test was set up in liquid, using natural must, and the capacity of 136 strains from the ITEM Collection to adsorb polyphenols onto their cell wall was subsequently evaluated by chemical assays on the resulting wines. A starter yeast population was then characterized with a total number of 136 strains (Table S1). The adopted procedure included the following steps: establishing of a population of starter yeasts extracted from the ITEM Collection, seedling on a YPD agar plate, preparing liquid cultures in YPD, inoculating selected must, initiating fermentation in triplicate, and assessing the total polyphenolic content (TPC). For inoculum preparation, 5 mL of Primitivo must was placed in sterile tubes with a 13 mL volume cap. Each thesis was individually inoculated with a yeast concentration of 0.4 × 10^6^ CFU/mL. Fermentations were carried out for 15 days, following which the determination of TPC was carried out for each sample using the Folin-Ciocalteu colorimetric assay and quantified as micrograms of gallic acid equivalent per mL (μgGAE/mL). 

Upon analyzing the concentrations of polyphenolic compounds in the wines produced by each yeast, we identified 29 strains exhibiting a TPC reduction of ≤50%, when compared to that detected in the utilized grape must. Additionally, the determination of the antioxidant capacity (TEAC), quantified as μmol of Trolox Equivalents per mL (μmolTE/mL), performed by ABTS^+^ colorimetric assay, was also reserved for the wines produced with the above 16 strains (Table 1). 

In view of the lower reduction of both TPC and TEAC, the strains 83, 84, 86, 99, 105, 106, 112, 113 and 135 were selected for the subsequent experiments. 

One liter of pasteurized Primitivo must was separately inoculated with 0.4 × 10^6^ CFU/mL of each of the above nine strains. Fermentations were conducted in triplicate and they took a regular course completing the alcoholic fermentation process in above eight days. The wines were analyzed by Fourier transform infrared spectrophotometry (FT-IR) for the main chemical parameters. Table 2 shows the results of the main physical-oenological and color parameters of the finished product, respectively. 

The ethanol amounts (g/100 mL) ranged from 11.97 (135) to 11.43 (86). The residual sugar contents determined for all the obtained wines was found in all wines below 2 g/L, a concentration consistent with a completed fermentation. The total acidity (TA) showed an amount varying from 7.50 g/L to 6.76 g/L, whereas the levels of volatile acidity (VA) ranged from 0.10 g/L to 0.14 g/L. As expected, all produced wine showed an acetic acid (VA) concentration < 0.2 g/L value. The nine selected yeast strain also produced a satisfactory quantity of glycerol, whose concentration ranged ranging from 7.75 to 6.38 g/L.

The nine resulting wines were then analyzed by assessing the concentration of total polyphenols (μgGAE/mL) by the Folin–Ciocalteu colorimetric assay and the antioxidant power (μmol TE/mL) through the TEAC assay (Table 3). As expected, the comparison of TPC and antioxidant activity in wines obtained by different yeast strain revealed a positive correlation. 

When inoculated into a larger volume of must, the nine yeast strains demonstrated a decreased capacity to adsorb wine polyphenol compounds on their external surfaces compared to the results reported in Table 1. Specifically, strains 6920, 9500, 9507, and 9508 exhibited a reduction rate of TPC and TEAC of less than 20%.

Table 4 reports the mean values and standard deviations of phenolic molecules identified and quantified in wines obtained by the nine selected yeast strains. Among the hydroxybenzoic acids, gallic acid was detected, while in the hydroxycinnamic acid derivatives, caffeoyl tartaric (caftaric) and *p*-coumaroyl tartaric (coutaric) acids were detected in the wine samples. Gallic acid showed higher amounts in wine produced by the 6993 strain. Caftaric acid was more concentrated in wine obtained from the 6993, 6920, 8766, 9507, and 9508 strains compared to the control strain. 

Coutaric acid showed a higher content in wines fermented by all strains, except 9501 and 9530, when compared to the control. The identified flavonol compound quercetin showed higher content in all samples in comparison to the control strain, except for the wine fermented by the 9530 strain. Catechin, belonging to the flavanol group of flavonoids, had a significantly higher amount only in wine fermented by 6993 strain.

Oenin was the non-acylated anthocyanin more abundant in all samples; its content was higher than control in wines fermented by the 8766 and 9500 strains. Trans-resveratrol, belonging to the stilbene class of polyphenols, showed a higher amount in wines fermented by the 6920, 9500, 9501, and 9507 strains compared to the control.

**Figure 1:**
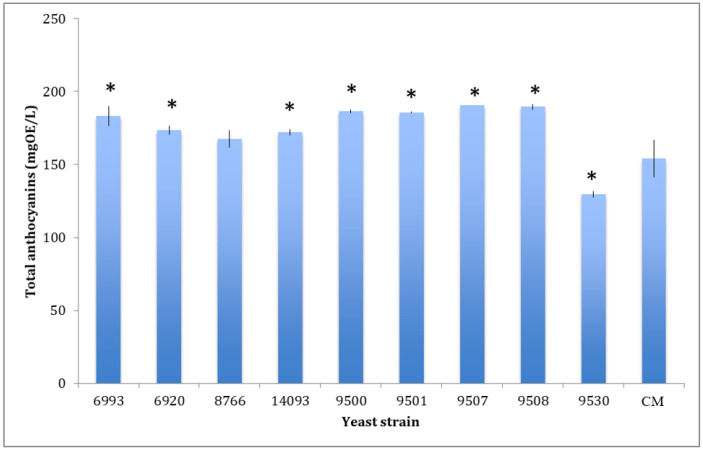
Comparison of total anthocyanin content in wines obtained by the nine-selected yeast and the commercial strain (CM). Data are mean ± S.D. and are representative of three different assays performed. OE = Oenin equivalent. Data were submitted to one-way analysis of variance (ANOVA), Tukey’s post hoc method was applied to establish differences between each yeast strain versus control. * indicates statistically significant differences (*p* < 0.05) between each yeast strain versus control.

The obtained data (Table 4) indicated that wines produced using the 9530 yeast strain starter contains significantly lower or comparable amounts of all identified polyphenol molecules compared to the control. The total anthocyanin content detected in each of the produced wines is shown in Figure 1. Total anthocyanin content was significantly higher than the control for all samples, except for the wine fermented by the 8766 strain, while the sample fermented by the 9530 strain exhibited a significantly lower anthocyanin concentration.

**Figure 2:**
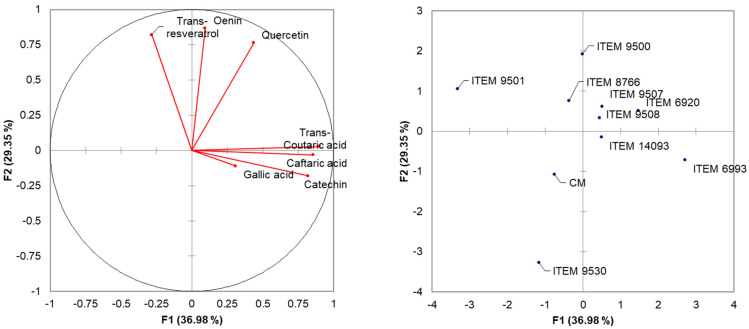
Principal Component Analysis (PCA) performed employing the data obtained by the HPLC analysis of the wines obtained using the nine selected strains.

HPLC data were subjected to Principal Component Analysis (PCA) based on Pearson correlation (*n* − 1) (Figure 2). The two principal components described the 66.33% of the total variance (36.98% and 29.35% for PC1 and PC2) of the phenolic acids data matrix: The analysis permitted the separation of yeast strains on the plane defined by two principal PCs. ITEM 9501, CM, and 9530, located to the left of PC2 result anti-correlated with the identified molecules, showing low concentrations. ITEM 9500 and ITEM 8766 cluster along the positive component of PC2 and show good correlation with trans-resveratrol, oenins, and quercetins. Along the positive component of PC1, ITEM 6993 is positively associated with trans-coutaric, caftaric, gallic acids and catechin. Finally, in the plane bounded by the positive components of the two PCs, we locate the cluster formed by ITEM 9507, 6920, 9508 and 9500 united by values closer to the mean value of all the molecules analyzed, showing profiles that are more balanced.

Based on the processing of the obtained data, strains ITEM6920, ITEM9500, ITEM9507, and ITEM9508 fulfilled the fundamental parameters required, in that: the grafted alcoholic fermentations had a consistent progression and duration (<10 days). FT-IR analysis confirmed the attainment of the expected ethanol content, with sugars being completely consumed (residual < 2 g/L) in the produced wines. Notably, these four demonstrated the absence of undesirable metabolites, particularly showing very low production of acetic acid (volatile acidity < 0.2 g/L). The wines produced showcased a reduction in the concentration of total polyphenols (≤20% TPC reduction) and a reduction in the antioxidant power (≤20% TEAC reduction). As a result, these four strains emerged as promising candidate starters for subsequent pilot and industrial-scale experiments set to be carried out in the winery. In evaluating the fermentation performance of these starter candidate strains, secondary fermentation products were detected and quantified in the nine wines by SPME-GC-MS analysis (Table 5).

**Figure 3:**
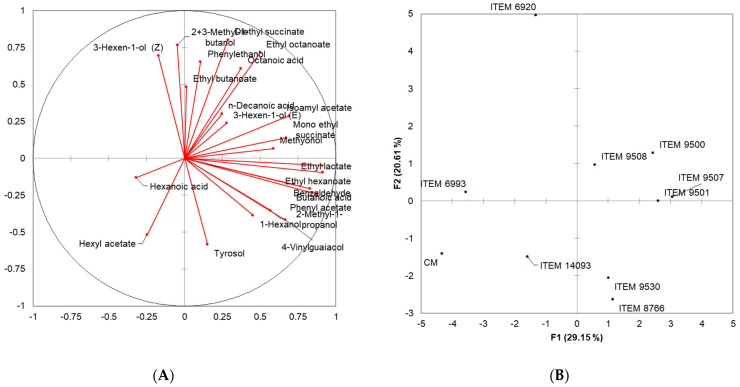
Two-dimensional Principal Component Analysis (PCA). Scoresplot (**A**) for the nine wines and (**B**) loading plot for Volatile Organic Compounds.

A total of 23 volatile substances belonging to the classes of alcohols, esters, aldehydes, phenols, and volatile acids were identified. Within the alcohol class, all samples presented higher values, ranging from 26.86 mg/L (ITEM 9530) to 51.57 mg/L (ITEM 9508). Notably, 2+3-methyl-1-butanol and phenylethanol emerged as the most abundant molecules among alcohols. The second class of molecules that quantitatively affect the complete volatile profile of the different samples is that of esters associated with fruity notes. The highest values were detected in ITEM 9507 (7.42 mg/L), ITEM 9500 (7.15 mg/L) ITEM 9501 (6.04 mg/L), ITEM 8766 (5.37 mg/L), and finally ITEM 6920 (5.03 mg/L). The presence of acetic acid was not detected, while an acidic component was identified, in quantities varying from 2.05 mg/L (CM) to 4.64 mg/L (ITEM 9508), due to the presence of butanoic, hexanoic, octanoic, and decanoic which contribute positively with notes of freshness. Furthermore, in order to identify the yeast strains that produce wines with the best volatile profiles, principal component analysis (PCA) was performed on the Pearson correlation matrix, on the concentrations of molecules detected by GC-MS (Figure 3).

The two bi-plots PC1 vs. PC2 showed the projection of the variables considered on the plane defined by the first and second principal components that explain 49.76% of the total variance.

Along the positive component of PC1 (29.15%), the strains ITEM 9500, ITEM 9501, ITEM 9507, and ITEM 9508 cluster in the same group for a positive correlation with n-decanoic acid, isoamyl acetate, monoethyl succinate, ethyl lactate, ethyl hexanoate, 3-hexen-1-ol (E), methyonol. On the other hand, along the positive component of PC2 (20.61), strain ITEM 6920 differs from the others for a greater content of phenylethanol, ethyl butanoate, octanoic acid, 2+3-methyl-1-butanol, ethyl octanoate and 3-hexen-1-ol (Z). All other strains were characterized by low concentrations of all identified volatiles.

## 4. Discussion

This investigation marks the first instance of analyzing an autochthonous starter culture collection, with the aim of selecting and characterizing strains exhibiting a low capacity to adsorb polyphenols onto their cell walls. This study establishes selection criteria based on the final concentration of total polyphenols and antioxidant capacity in wine samples obtained at the end of the various experimental phases. Previous studies analyzed polyphenolic variation was by evaluating parameters such as color intensity and shade of the yeast colonies post-fermentation, quantified using empirical criteria [37]. We developed a selection protocol based on the evaluation of the residual polyphenolic component and antioxidant activity in wines fermented with the strains under analysis, employing Folin–Ciocalteu and TEAC assays.

A total of 136 *S. cerevisiae* starter strains were chosen from the CNR ISPA microbial collection, and their capacity to enhance the polyphenolic content in produced wines was evaluated by laboratory-scale fermentation analysis.

The results indicated that all strains decreased the concentration of polyphenolic compounds and, consequently, the antioxidant power. The top-performing strains, demonstrating a lower capacity to adsorb polyphenols, were selected and characterized throughout the various phases of the study. It is known from the literature that yeast’s enzymes (pectinase and glycosidases) may affect polyphenolic extraction, modifying the chemical structure of glycosylated phenolic molecules [38]. Moreover, polyphenols interact with yeast by binding to cell wall mannoprotein [9]. The observed decrease in TPC and antioxidant activity is likely related to these interaction mechanisms between polyphenols and yeasts. These interaction mechanisms are related also to the variation of the polyphenolic profile described in Table 4 as hydrolysis process during winemaking alters the rate between free and glycoside forms of phenolic compounds. 

Our results confirmed the data obtained by Brandolini et al. [39], who investigated the properties of wines produced by separate inoculation of *S. cerevisiae* strains into the same must. The study highlighted strain-specific abilities to differentially adsorb polyphenols during the vinification process. Similar results were obtained by Kostadinović et al. [40] on Vranec and Merlot wines in Macedonia, emphasizing the strain-specific influence on the concentration of trans-resveratrol and antioxidant activities. The use of different yeast strains also demonstrated varying polyphenolic content in Pinot Noir wines [13]. Indeed, five different yeast starters were tested in several vinifications, in which the *S. cerevisiae* strain RC212 was able to significantly increase the concentrations of total pigments, anthocyanins and tannins. Carrascosa and associates [41] demonstrated that different yeast strains were able to produce Albariño wines characterized by a specific polyphenolic composition. The above results were further confirmed by a recent report [21], in which an unequivocal correlation between the yeast used to promote the fermentation process and the chemical profile of the wine was recognized, thus underlining the strain-specific abilities of yeasts to modify the color and polyphenolic composition of the final product.

Furthermore, a recent study identified yeast starter cultures capable of improving the quality of wine produced from the Italian red cultivar ‘Gaglioppo’, a cultivar with reduced anthocyanin synthesis [42]. Again, the evidence obtained further highlighted the specific ability of some strains to modify the final amounts of total anthocyanin, total polyphenols and total tannins.

Recently, Grieco et al. [43] highlighted a positive role of indigenous yeast cultures in improving polyphenol content across the industrial production of Negroamaro and Primitivo wines. Statistical analysis showed that the use of indigenous strains increased the concentrations of several classes of polyphenols in the wines produced compared to wines made with a commercial strain.

It can be asserted that the different ability shown by our yeast strains in complexing phenolic compounds on their cell wall is a strain-specific property [44,45]. The evidence that the four yeast starter-culture identified during this study showed the capacity to adsorb the least amount of all types of anthocyanin is consistent with the evidence demonstrated by previous studies in Spain [10], France [45] and Italy [46].

Therefore, it is imperative to consider the adsorption ability of phenolic compounds during selective procedures for yeasts starter culture in wine production [9].

Nevertheless, it is worthy to note the effect of scale comparing the differences between the performance of the selected strains in reducing TPC between the micro-scale and laboratory-scale vinification. This finding is in agreement with our previous investigation [47], where we explored the oenological significance of winemaking scales in evaluating the contribution of new starter cultures to the chemical profile of produced wine. 

As regards the volatolomic aspect that influences the sensorial quality, our results confirm that the ITEM 6920-9500-9507-9508 strains contribute to a balanced volatile profile, mainly characterized by secondary fermentation products. In particular, ethyl ester concentrations were influenced by the yeast strain, fermentation temperature, degree of aeration, and sugar content. Both ethyl esters and acetate esters have a key importance in the overall aroma of wine, contributing positive sensory notes like sweet-fruity, grape smell, and sweet balsamic [48,49]. Concerning esters, the mentioned strains showed an increased ester production ofisoamyl acetate, ethyl hexanoate, diethyl succinate, phenyl acetate and mono ethyl succinate. Similarly, among higher alcohols, 2-phenylethanol contributes a floral (pink) aroma [50], but an excess concentration above 300 mg/L would impart a strong and pungent odor and taste [51]. In our study, the use of selected yeast strains allowed us to obtain higher alcohols in concentrations lower than the critical threshold value, with a positive contribution to the sensorial profile, and phenylethanol values higher than the perception threshold (10 mg/L) [52,53] except for ITEM 9501 and 14093. Finally, the contribution of the volatile acid fraction also appears positive. Indeed, fatty acids, produced during fermentation, constitute an important group of aromatic compounds that can provide fruity, cheesy, fatty, and rancid notes. In this case, the quantified fatty acids had levels lower than their perception threshold.

By processing the data obtained at the end of the various experimental phases, we were able to identify four strains (ITEM6920, ITEM9500, ITEM9507, and ITEM9508) capable of producing wine characterized by a higher concentration of total polyphenols, an enhanced antioxidant capacity, and the absence of undesirable metabolites.

## 5. Conclusions

The results of this study suggest that the exploitation of autochthonous yeast strains can enhance the antioxidant activity and the amount of the phenolic compounds in the produced wine. These autochthonous microbial resources can be denoted as “antioxidant positive strain” [43] since they were able to increase both the health promoting [1] and aromatic properties of wine in synergy with the use of innovative technological processes [54]. Taken together, our finding emphasize the relevance of developing and applying innovative biotechnological approaches to enhance the presence in wine of molecules with potential benefits to human health, thus improving the ‘functional parameters’ and the overall quality of the final product. Ongoing studies are exploring the industrial application of these four autochthonous strains as starter cultures for the large-scale production of typical red wines.

## Figures and Tables

**Table 1:** Analysis of Total Phenolic Content (TPC) and Trolox Equivalent Antioxidant Capacity (TEAC) of wines obtained from selected yeasts based on the least reduction of the polyphenolic component. The results of the nine best-performing strains were reported in bold.

ID	Strain	TPC (µgGAE/mL)	%TPC Reduction	TEAC (nmolTE/mL)	%TEAC Reduction
20	6978	607.60 ± 5.26	−48%	47.63 ± 0.97	−46%
63	8754	634.99 ± 5.43	−46%	63.70 ± 2.43	−27%
64	8744	623.57 ± 7.46	−47%	54.24 ± 3.01	−38%
69	8766	601.61 ± 5.76	−48%	63.50 ± 2.09	−27%
70	8767	612.87 ± 8.09	−48%	59.98 ± 1.54	−31%
71	8769	620.08 ± 5.73	−47%	72.75 ± 2.22	−17%
74	8772	627.44 ± 5.90	−46%	74.66 ± 2.00	−15%
77	8775	609.70 ± 8.78	−48%	72.22 ± 1.76	−17%
78	8776	651.59 ± 4.94	−44%	73.20 ± 1.05	−16%
80	8778	605.66 ± 3.76	−48%	61.80 ± 0.97	−29%
81	8779	616.54 ± 3.87	−47%	55.76 ± 0.90	−36%
82	8780	639.91 ± 5.85	−45%	63.57 ± 1.02	−27%
**83**	**6993**	**785.14 ± 8.47**	**−33%**	**64.04 ± 1.00**	**−27%**
**84**	**6920**	**789.55 ± 9.76**	**−32%**	**64.81 ± 0.99**	**−26%**
**86**	**8766**	**699.95 ± 6.73**	**−40%**	**61.60 ± 2.37**	**−30%**
87	6977	607.68 ± 2.38	−48%	63.23 ± 1.76	−28%
89	8795	612.56 ± 5.77	−48%	59.79 ± 1.43	−32%
90	17,292	626.68 ± 4.38	−46%	56.06 ± 0.98	−36%
91	17,293	636.65 ± 5.65	−45%	66.38 ± 1.76	−24%
92	9502	608.41 ± 2.90	−48%	64.86 ± 2.02	−26%
96	9531	643.67 ± 3.35	−45%	64.81 ± 1.74	−26%
98	1407	629.36 ± 4.84	−46%	62.40 ± 1.64	−29%
**99**	**14,093**	**746.44 ± 2.37**	**−36%**	**64.79 ± 1.23**	**−26%**
**105**	**9500**	**786.23 ± 5.74**	**−33%**	**62.95 ± 2.09**	**−28%**
**106**	**9501**	**755.92 ± 6.68**	**−35%**	**57.90 ± 0.98**	**−34%**
107	9502	607.47 ± 2.64	−48%	59.17 ± 0.78	−32%
**112**	**9507**	**766.47 ± 5.89**	**−34%**	**68.38 ± 2.20**	**−22%**
**113**	**9508**	**779.70 ± 3.65**	**−33%**	**65.78 ± 1.43**	**−25%**
**135**	**9530**	**729.32 ± 5.74**	**−38%**	**58.66 ± 0.98**	**−33%**
**Must**		1167.45 ± 8.46		87.49 ± 3.01	

**Table 2:** Analysis by FT-IR assay of the main chemical parameters of wines produced from the nine selected *S. cerevisiae* strains and the commercial yeast strain (CM).

Strain	Ethanol	Sugars	TA	VA	Malic Acid	Lactic Acic	Glycerol
6993	11.45 ± 0.023	1.6 ± 0.034	7.11 ± 0.008	0.14 ± 0.013	2.58 ± 0.005	0.15 ± 0.004	6.38 ± 0.055
6920	11.58 ± 0.004	1.34 ± 0.059	7.14 ± 0.056	0.07 ± 0.15	2.64 ± 0.002	0.08 ± 0.001	6.54 ± 0.088
8766	11.43 ± 0.061	1.28 ± 0.334	7.41 ± 0.022	0.15 ± 0.008	2.61 ± 0.025	0.2 ± 0.057	7.03 ± 0.028
14093	11.47 ± 0.004	1.39 ± 0.105	7.11 ± 0.03	0.11 ± 0.003	2.56 ± 0.035	0.07 ± 0.062	6.68 ± 0.055
9500	11.75 ± 0.033	1.35 ± 0.136	7.24 ± 0.006	0.15 ± 0.024	2.49 ± 0.039	0.18 ± 0.017	7.21 ± 0.116
9501	11.58 ± 0.014	1.40 ± 0.130	6.76 ± 0.013	0.11 ± 0.004	2.05 ± 0.012	0.39 ± 0.007	7.48 ± 0.128
9507	11.74 ± 0.019	1.48 ± 0.160	7.25 ± 0.017	0.16 ± 0.011	2.54 ± 0.016	0.18 ± 0.059	6.97 ± 0.09
9508	11.96 ± 0.028	1.67 ± 0.073	7.5 ± 0.039	0.13 ± 0.002	2.67 ± 0.016	0.12 ± 0.057	7.19 ± 0.046
9530	11.97 ± 0.004	1.24 ± 0.055	7.97 ± 0.022	0.10 ± 0.018	2.83 ± 0.055	0.33 ± 0.022	7.75 ± 0.033
CM	8.14 ± 0.012	39.51 ± 0.014	7.48 ± 0.023	0.14 ± 0.002	2.47 ± 0.047	0	5.88 ± 0.043

TA, total acidity. VA, volatile acidity. The ethanol concentration is expressed as g/100 mL. The other values are expressed as g/L. CM, commercial starter control. Initial sugar content in the must was 21° Brix.

**Table 3:** Analysis of Total Phenolic Content (TPC) and Trolox Equivalent Antioxidant Capacity (TEAC) of wines obtained from the nine selected yeasts and the commercial strain (CM).

ITEM	TPC (µgGAE/mL)	TPC % Reduction	TEAC (μmolTE/mL)	TEAC % Reduction
6993	600.81 ± 1.10 ^dc^	−32%	4.49 ± 0.06 ^cd^	−21%
**6920**	**701.70** ± 0.60 ^b^	**−20%**	**4.56** ± 0.16 ^cd^	**−19%**
8766	611.59 ± 37.60 ^cde^	−30%	4.38 ± 0.02 ^cd^	−22%
14093	630.71 ± 0.76 ^bcde^	−28%	4.44 ± 0.07 ^cd^	−21%
**9500**	**699.74** ± 1.41 ^bc^	**−20%**	**4.62** ± 0.02 ^bc^	**−18%**
9501	551.04 ± 20.00 ^ef^	−37%	4.22 ± 0.14 ^d^	−25%
**9507**	**712.13** ± 2.49 ^b^	**−19%**	**4.51** ± 0.02 ^cd^	**−20%**
**9508**	**801.64** ± 53.60 ^a^	**−9%**	**4.97** ± 0.20 ^b^	**−12%**
9530	639.39 ± 26.60 ^bcd^	−27%	4.43 ± 0.04 ^cd^	−22%
CM	502.04 ± 0.13 ^f^	−43%	3.12 ± 0.02 ^f^	−44%
Must	876.57 ± 5.15 ^a^	//	5.66 ± 0.04 ^a^	//

The reported values are means (*n* = 3) ± standard deviation. Mean that do not share a letter are significantly different. The results of the four best-performing strains were reported in bold. Data were submitted to one-way analysis of variance (ANOVA), Tukey’s post hoc method was applied to establish differences between each yeast strain versus control. Different letters indicate statistically significant differences (*p* < 0.05).

**Table 4:** Polyphenols content (mg/L) of wines obtained by the nine selected yeasts and the commercial strain (CM).

**Strain**	**Quercetin**	**Gallic Acid**	**Catechin**	**Oenin**	**Trans-Resveratrol**	**Trans-Coutaric Acid**	**Caftaric Acid**
	mg/L						
6993	2.857 ± 0.091 ^b^	32.872 ± 0.486 ^b^	58.207 ± 2.593 ^b^	115.251 ± 1.096 ^b^	1.412 ± 0.118 ^a^	14.097 ± 0.113 ^b^	52.723 ± 0.900 ^b^
6920	2.433 ± 0.152 ^b^	30.381 ± 0.549 ^a^	49.160 ± 4.002 ^a^	120.966 ± 0.643 ^a^	2.766 ± 0.097 ^b^	16.039 ± 0.214 ^b^	56.158 ± 0.751 ^b^
8766	2.369 ± 0.391 ^b^	25.187 ± 0.590 ^b^	44.896 ± 0.375 ^a^	134.179 ± 1.729 ^b^	1.710 ± 0.069 ^a^	9.447 ± 0.157 ^b^	43.842 ± 0.614 ^b^
14093	2.456 ± 0.295 ^b^	30.530 ± 0.344 ^a^	50.545 ± 3.924 ^a^	121.750 ± 0.924 ^a^	1.721 ± 0.016 ^a^	8.806 ± 0.059 ^b^	42.246 ± 0.198 ^a^
9500	3.375 ± 0.441 ^b^	27.015 ± 0.427 ^b^	48.366 ± 0.335 ^a^	131.042 ± 2.551 ^b^	3.085 ± 0.067 ^b^	8.341 ± 0.052 ^b^	42.143 ± 0.332 ^a^
9501	2.132 ± 0.391 ^b^	29.516 ± 0.673 ^a^	42.259 ± 3.506 ^a^	121.123 ± 3.725 ^a^	3.528 ± 0.088 ^b^	0.563 ± 0.000 ^b^	3.130 ± 0.113 ^b^
9507	2.184 ± 0.115 ^b^	24.349 ± 0.179 ^b^	44.707 ± 0.086 ^a^	127.983 ± 1.007 ^a^	2.139 ± 0.446 ^b^	14.093 ± 0.046 ^b^	60.821 ± 1.713 ^b^
9508	2.902 ± 0.189 ^b^	26.484 ± 0.875 ^b^	47.336 ± 0.815 ^a^	125.542 ± 3.056 ^a^	1.442 ± 0.028 ^a^	8.730 ± 0.138 ^b^	54.666 ± 1.254 ^b^
9530	0.671 ± 0.101 ^a^	26.277 ± 0.834 ^b^	46.072 ± 0.704 ^a^	92.511 ± 0.632 ^b^	0.515 ± 0.007 ^b^	7.157 ± 0.154 ^a^	37.062 ± 0.796 ^b^
CM	0.677 ± 0.059 ^a^	29.559 ± 0.009 ^a^	47.319 ± 3.573 ^a^	124.157 ± 0.709 ^a^	1.565 ± 0.079 ^a^	6.832 ± 0.017 ^a^	40.754 ± 0.010 ^a^

CM, commercial yeast strain. Data are mean ± S.D. and are representative of three different assays performed. Data were submitted to one-way analysis of variance (ANOVA), Tukey’s post hoc method was applied to establish differences between each yeast strain versus control. Different letters indicate statistically significant differences (*p* < 0.05).

**Table 5:** Volatile Organic Compounds detected in wine samples.

Volatiles (mg/L)	ITEM 6993	*±SD*	ITEM 6920	*±SD*	ITEM 8766	*±SD*	ITEM 14093	±*SD*	ITEM 9500	*±SD*	ITEM 9501	*±SD*	ITEM 9507	*±SD*	ITEM 9508	*±SD*	ITEM 9530	*±SD*	CM	*±SD*	*Statistical Significance*
**Esters**																					
Ethyl butanoate	0.110	*0.050*	0.715	*0.210*	0.142	*0.060*	0.150	0.06	0.760	*0.180*	0.450	*0.170*	0.650	*0.170*	0.611	*0.017*	0.204	*0.042*	0.940	*0.140*	***
Isoamyl acetate	0.722	*0.210*	1.442	*0.620*	1.197	*0.340*	0.170	0.05	1.820	*0.640*	1.080	*0.023*	2.050	*0.820*	0.610	*0.032*	1.450	*0.260*	0.514	*0.095*	*
Ethyl hexanoate	0.149	*0.060*	0.158	*0.050*	0.492	*0.080*	0.127	0.04	0.514	*0.120*	0.430	*0.110*	0.610	*0.210*	0.450	*0.120*	0.340	*0.070*	0.110	*0.040*	**
Hexyl acetate	nd		nd		0.093	*0.014*	0.076	0.012	0.095	*0.014*	0.056	*0.014*	0.066	*0.014*	0.053	*0.011*	0.087	*0.015*	0.250	*0.074*	***
Ethyl lactate	nd		0.092	*0.014*	0.235	*0.092*	0.217	0.051	0.460	*0.080*	0.560	*0.080*	0.411	*0.130*	0.405	*0.210*	0.326	*0.066*	0.042	*0.011*	**
Ethyl octanoate	0.079	*0.020*	0.570	*0.070*	0.214	*0.014*	0.156	0.07	0.440	*0.130*	0.380	*0.110*	0.278	*0.080*	0.316	*0.080*	0.320	*0.080*	0.147	*0.040*	**
Diethyl succinate	0.123	*0.060*	0.750	*0.130*	0.341	*0.032*	0.188	0.012	0.470	*0.170*	0.270	*0.070*	0.310	*0.070*	0.310	*0.040*	0.167	*0.030*	0.122	*0.040*	***
Phenyl acetate	0.353	*0.140*	0.207	*0.080*	1.269	*0.510*	1.160	0.33	1.650	*0.910*	1.770	*0.640*	2.110	*0.940*	0.850	*0.170*	0.950	*0.210*	0.281	*0.104*	*
Mono ethyl succinate	0.654	*0.210*	1.096	*0.340*	1.390	*0.660*	0.860	0.24	0.94	*0.18*	1.04	*0.15*	0.930	*0.360*	0.910	*0.310*	0.870	*0.140*	0.142	*0.080*	ns
Total Esters	**2.19**		**5.03**		**5.37**		**3.10**		**7.15**		**6.04**		**7.42**		**4.52**		**4.71**		**2.55**		
**Alcohols**																					
2-Methyl-1-propanol	0.20	*0.06*	0.76	*0.12*	2.54	*0.57*	0.95	0.2	1.25	*0.04*	0.92	*0.17*	1.62	*0.35*	0.47	*0.06*	0.88	*0.20*	0.55	*0.14*	***
3-Methyl-1-butanol	27.15	*2.35*	28.72	*5.04*	16.12	*4.05*	18.93	5.1	24.76	*5.11*	16.90	*4.32*	21.88	*4.15*	31.16	*4.28*	11.87	*3.17*	14.22	*2.07*	**
1-Hexanol	1.06	*0.04*	0.54	*0.08*	0.82	*0.12*	0.95	0.18	1.37	*0.34*	1.78	*0.65*	1.96	*0.62*	1.26	*0.33*	2.87	*0.64*	1.25	*0.04*	**
3-Hexen-1-ol (E)	0.17	*0.02*	0.33	*0.03*	0.18	*0.04*	0.26	0.08	0.27	*0.07*	0.44	*0.10*	0.22	*0.07*	0.31	*0.01*	0.35	*0.08*	0.28	*0.11*	ns
3-Hexen-1-ol (Z)	0.17	*0.05*	0.55	*0.08*	0.19	*0.06*	0.25	0.06	0.18	*0.06*	0.34	*0.07*	0.21	*0.04*	0.17	*0.05*	0.12	*0.04*	0.27	*0.15*	**
Methyonol	0.29	*0.07*	0.36	*0.10*	0.60	*0.17*	0.17	0.03	0.55	*0.10*	0.37	*0.08*	0.35	*0.03*	0.22	*0.04*	0.28	*0.06*	0.150	*0.04*	**
Phenylethanol	19.23	*3.520*	18.607	*4.070*	11.156	*3.070*	9.450	1.51	19.400	*3.610*	8.550	*2.170*	20.140	*5.140*	17.980	*3.910*	10.500	*2.180*	10.240	*1.82*	*
Total Alcohols	**48.27**		**49.87**		**31.61**		**30.96**		**47.78**		**29.30**		**46.38**		**51.57**		**26.86**		**26.96**		
**Aldehydes**																					
Benzaldehyde	0.10	*0.0300*	0.330	*0.0700*	0.420	*0.1100*	0.92	0.24	0.56	*0.14*	1.10	*0.03*	1.15	*0.07*	0.85	*0.18*	0.870	*0.1700*	0.25	*0.06*	***
**Volatiles phenol**																					
4-Vinylguaiacol	1.480	*0.550*	nd		4.45	*0.95*	1.76	0.61	2.55	*0.61*	2.87	*0.51*	3.98	*0.72*	5.38	*0.94*	4.10	*0.92*	0.65	*0.17*	***
Tyrosol	0.995	*0.370*	nd		5.53	*1.04*	nd		0.36	*0.08*	0.77	*0.18*	0.95	*0.18*	nd		1.76	*0.34*	1.040	*0.320*	***
Total Volatile Phenols	**2.47**				**9.97**		**1.76**		**2.91**		**3.64**		**4.93**		**5.38**		**5.86**		**1.69**		
**Volatile acids**																					
Butanoic acid	nd		nd		0.26	*0.06*	0.41	0.07	0.55	*0.11*	0.63	*0.25*	0.41	*0.07*	0.35	*0.08*	0.36	*0.07*	nd		***
Hexanoic acid	1.63	*0.27*	0.83	*0.18*	1.18	*0.04*	0.87	0.18	0.77	*0.21*	0.93	*0.18*	0.88	*0.13*	1.34	*0.33*	0.95	*0.16*	0.880	*0.1700*	*
Octanoic acid	1.74	*0.14*	1.71	*0.42*	0.63	*0.17*	0.94	0.24	1.45	*0.43*	1.76	*0.34*	1.88	*1.04*	1.33	*0.17*	1.36	*0.35*	0.620	*0.2300*	ns
*n*-Decanoic acid	0.43	*0.08*	0.52	*0.17*	0.34	*0.05*	nd		0.56	*0.07*	0.87	*0.21*	0.55	*0.14*	1.62	*0.54*	0.41	*0.11*	0.550	*0.1600*	**
Total Volatile Acids	**3.79**		**3.05**		**2.41**		**2.22**		**3.33**		**4.19**		**3.72**		**4.64**		**3.08**		**2.05**		

nd: not detected; sd: standard deviation; significant differences * *p* < 0.05; ** *p* < 0.01; *** *p* < 0.001.

## Data Availability

Data is contained within the article or Supplementary Materials.

## Supplementary Materials

The following supporting information can be downloaded at: https://www.mdpi.com/article/10.3390/foods13020312/s1, Table S1: List of yeast strains used.
