# Supplementary material for: Yeast Starter Culture Identification to Produce of Red Wines with Enhanced Antioxidant Content
Source: Foods. 2024 Jan 18;13(2):312. doi: 10.3390/foods13020312 (PMC10815507; doi:10.3390/foods13020312)
Supplement: Supplementary file 1 [file foods-13-00312-s001.zip › foods-2809358-supplementary.pdf]

Table S1. List of yeast strains used.

ID	Denomination	ITEM code
**1**	1TTA5	6951
**2**	2TTA6	6952
**3**	3TTA8	6953
**4**	4TTA9	6954
**5**	6TTA13	6956
**6**	9TTA22	6957
**7**	11TTA27	6958
**8**	17TTA38	6962
**9**	18TTA47	6964
**10**	25TTB16	6955
**11**	26TTB21	6968
**12**	31TTB33	6969
**13**	34TTB46	6971
**14**	35TTB47	6972
**15**	36TTB50	6963
**16**	1MMA2	6973
**17**	2MMA4	6974
**18**	3MMA11	6975
**19**	8MMA26	6977
**20**	10MMA41	6978
**21**	11MMA50	6979
**22**	13MMB5	6980
**23**	14MMB6	6981
**24**	24MMB25	6984
**25**	25MMB27	6985
**26**	27MMB31	6986
**27**	33MMB47	6989
**28**	4CA93	6904
**29**	4CA107	6905
**30**	4CA116	6907
**31**	4CB2	6908
**32**	4CB27	6909
**33**	4CB31	6910
**34**	4CB47	6911
**35**	4CB64	6912
**36**	4CB77	6914
**37**	4CC4	6916
**38**	4CC104	6917
**39**	5CA62	6918
**40**	5CB10	6919
**41**	5CC23	6921
**42**	5CC76	6922
**43**	5CC129	6923
**44**	KA2	6924
**45**	KA4	6925
**46**	KA8	6926
**47**	KA33	6927
**48**	KA37	6928

**49**	KA46	6929
**50**	KB6	6930
**51**	KB10	6931
**52**	KB13	6932
**53**	KB26	6937
**54**	KC13	6945
**55**	KC16	6946
**56**	GA3G2	8743
**57**	GA12G4	8746
**58**	GA25G6	8747
**59**	GA29G8	8748
**60**	GA34G11	8749
**61**	GA36G12	8742
**62**	GB26G27	8752
**63**	GB49G35	8754
**64**	GA6G37	8744
**65**	GA8G38	8745
**66**	GA47G44	8750
**67**	GB3G45	8751
**68**	GB31G49	8753
**69**	MA2M1	8766
**70**	MA4M2	8767
**71**	MA6M4	8769
**72**	MA9M5	8770
**73**	MA12M6	8771
**74**	MA19M8	8772
**75**	MA27M11	8773
**76**	MA28M12	8774
**77**	MA37M16	8775
**78**	MB1M18	8776
**79**	MB9M19	8777
**80**	MB13M21	8778
**81**	MB19M25	8779
**82**	MB20M26	8780
**83**	B1/TORZ	6993
**84**	C19	6920
**85**	TOM14	8760
**86**	EM1/MN1	8766
**87**	8M	6977
**88**	14M	6981
**89**	1M4	8795
**90**	P25	17292
**91**	P28	17293
**92**	S8	9502
**93**	S39/S33	9518
**94**	S40/SS4	9519
**95**	S41/SS5	9520
**96**	S71/SS35	9531
**97**	NC103	14061
**98**	NU32	14077
**99**	PR12	14093
**100**	PMR01	17294

**101**	E4	-
**102**	SA6	9497
**103**	SA18	9498
**104**	SA27	9499
**105**	SA23	9500
**106**	SA1	9501
**107**	SA41	9502
**108**	SA37	9503
**109**	SA9	9504
**110**	SA28	9505
**111**	SB16	9506
**112**	SB21	9507
**113**	SB43	9508
**114**	SB46	9509
**115**	SB35	9510
**116**	SB8	9511
**117**	SB30	9512
**118**	SB24	9513
**119**	SB48	9514
**120**	SB52	9515
**121**	SSA40	9516
**122**	SSA44	9517
**123**	SSA48	9518
**124**	SSA13	9519
**125**	SSA22	9520
**126**	SSA6	9521
**127**	SSA7	9522
**128**	SSA20	9523
**129**	SSA1	9524
**130**	SSB27	9525
**131**	SSB19	9526
**132**	SS23	9527
**133**	SS25	9528
**134**	SS28	9529
**135**	SS33	9530
**136**	SS35	9531
